# *Trypanosoma cruzi* infection induces DNA double-strand breaks and activates DNA damage response pathway in host epithelial cells

**DOI:** 10.1038/s41598-024-53589-w

**Published:** 2024-03-04

**Authors:** Raul Alexander Gonzáles-Córdova, Thamires Rossi dos Santos, Camila Gachet-Castro, Johnathan Andrade Vieira, Lays Adrianne Mendonça Trajano-Silva, Elza Tiemi Sakamoto-Hojo, Munira Muhammad Abdel Baqui

**Affiliations:** 1 Department of Cellular and Molecular Biology and Pathogenic Bioagents, Ribeirão Preto Medical School, University of São Paulo-USP, Ribeirão Preto, 14049-900 Brazil; 2 Department of Genetics, Ribeirão Preto Medical School, University of São Paulo-USP, Ribeirão Preto, 14049-900 Brazil; 3 Department of Biology, Faculty of Philosophy Sciences and Letters at Ribeirão Preto, University of São Paulo, São Paulo, 14040-901 Brazil

**Keywords:** Parasite host response, DNA damage response

## Abstract

*Trypanosoma cruzi*, the etiological agent of Chagas disease, invades many cell types affecting numerous host-signalling pathways. During the *T. cruzi* infection, we demonstrated modulations in the host RNA polymerase II activity with the downregulation of ribonucleoproteins affecting host transcription and splicing machinery. These alterations could be a result of the initial damage to the host DNA caused by the presence of the parasite, however, the mechanisms are not well understood. Herein, we examined whether infection by *T. cruzi* coincided with enhanced DNA damage in the host cell. We studied the engagement of the DNA damage response (DDR) pathways at the different time points (0–24 h post-infection, hpi) by *T. cruzi* in LLC-MK2 cells. In response to double-strand breaks (DSB), maximum phosphorylation of the histone variant H2AX is observed at 2hpi and promotes recruitment of the DDR p53-binding protein (53BP1). During *T. cruzi* infection, Ataxia-telangiectasia mutated protein (ATM) and DNA-PK protein kinases remained active in a time-dependent manner and played roles in regulating the host response to DSB. The host DNA lesions caused by the infection are likely orchestrated by the non-homologous end joining (NHEJ) pathway to maintain the host genome integrity.

## Introduction

Chagas disease (CD), or American Trypanosomiasis, is a chronic neglected tropical disease (NTD) caused by the flagellate protozoan *T. cruzi*^1^. In Latin America, 21 countries are considered endemic for CD, with more than 10,000 deaths per year attributed to this disease^2^. CD affects ~ 8 million individuals worldwide, with ~ 70 million living at risk of infection due to the change in migratory dynamics in recent years due to the global economic crisis^2–4^.

*Trypanosoma cruzi* has a complex heteroxenous life cycle involving both invertebrate and vertebrate hosts. The well-defined developmental cycle in mammals begins following infection by trypomastigotes that invade immune and non-immune host cells, subsequently differentiating into the replicative amastigote forms. Intracellular *T. cruzi* can survive and proliferate in different metabolic environments by modulating the host cell^5,6^, allowing *T. cruzi* to evade clearance by the host immune system^7,8^, for example, suppressing oxidative activity in phagocytic cells^6^, preventing apoptosis in cardiac cells^9^, and inducing senescence in fibroblasts^10^. In contrast, infection by *T. cruzi* triggers a series of heterogeneous host cell responses, including an acute inflammatory response^11^, increased lipid synthesis^12^, decreased antioxidant response^11^, cytoskeleton modifications^11,13,14^, neuronal damage^15^, increased oxidative phosphorylation^16^, and changes to hormone and protein synthesis^17^.

More recently, we have shown that *T. cruzi* modulated transcription and splicing machinery within the host cell, causing alterations in the host RNA polymerase II activity with downregulation of the ribonucleoproteins associated with mRNA transcription and hijacking the auxiliary splicing factor U2AF35^18^. It has been reported that RNA Pol II can be rapidly blocked or degraded due to DNA damage, thus preventing the transcription complex from accessing any gene^19^. We also report the proximity of the parasite to the host cell nucleus^18^. Thus, the interaction between *T. cruzi* and the host cell nucleus reinforces the idea that *T. cruzi* modulates several host responses and may affect the integrity of the host cell DNA.

Cells routinely experience damage to their DNA from various exogenous and endogenous sources^20^. Most endogenous DNA damage comes from the cell metabolic activity, where DNA chemically reacts with water and reactive oxygen species (ROS). Exogenous DNA damage occurs when environmental, physical, and chemical agents damage DNA, such as UV and ionizing radiations, alkylating agents, and cross-linking agents. In addition to the classical agents that cause DNA damage, pathogenic microbes such as viruses, bacteria, and parasites can develop strategies to alter, subvert and manipulate host DNA damage and repair pathways during infection to survive and persist infection^21–30^. Regarding trypanosomatids, there is limited information about their impact on host DNA. Thus, to maintain genome integrity, eukaryotic cells have evolved a series of interconnected networks that respond to and resolve DNA injuries^31^. The DNA damage response (DDR) pathway is highly conserved in eukaryotes. It comprises several key proteins and can be subdivided into three stages: damage recognition, signal amplification, and damage repair^31–33^. DNA damage is recognised by structures that are already part of the histone core, such as H2AX, or by proteins that bind to aberrant DNA structures, such as PARP, the Mre11-Rad50-Nbs1 complex, the Rad9-Rad1-Hus1 complex, RPA, ATRIP, and PCNA^32–36^.

At the apex of the DDR pathway in most eukaryotes, two protein kinases act as key orchestrators; Ataxia Telangiectasia Mutated (ATM) and Ataxia Telangiectasia and Rad3-related (ATR)^37^. Both ATM and ATR belong to the phosphatidylinositol 3-kinase (PI3K)-related kinase (PIKK) family and become activated in response to DNA damage^38^. In higher eukaryotes, the DNA-PK protein, which also belongs to the PIKK family protein, is part of the group of orchestrator proteins and mediates the response to DNA damage^38^. Also, DNA-PK is part of a group of DNA repair proteins as well as DNA repair polymerases, DNA ligases, Rad51, and XRCC4^20^. 53BP1, MDC1, Ku70/80, BRCA1/2, and Artemis proteins act as mediators among sensor, transducer, and repair proteins in the DDR pathway^34,39^. The correct orchestration of this pathway is crucial to maintain the genome integrity and allows cells to continue cell cycle progression and survive. It has been reported that infection by *T. cruzi* causes alterations in the host chromatin, affecting the host genome stability^40,41^. However, the activation dynamics of the key proteins of the DDR pathway have yet to be well studied during *T. cruzi* infection.

Here, we report in LLC-MK2 non-phagocytic epithelial cells that *T. cruzi* infection induces host DNA damage in the first 24hpi, with 12hpi being the highest number of DNA breaks. At the same time, a cascade of various DDR pathway proteins is activated in the host and is orchestrated by phosphorylation dynamics in a time-dependent manner. In this time window, DSB are repaired by the NHEJ pathway to protect the genome integrity.

## Results

### Host DNA breaks are observed during *Trypanosoma cruzi* infection

**Figure 1:**
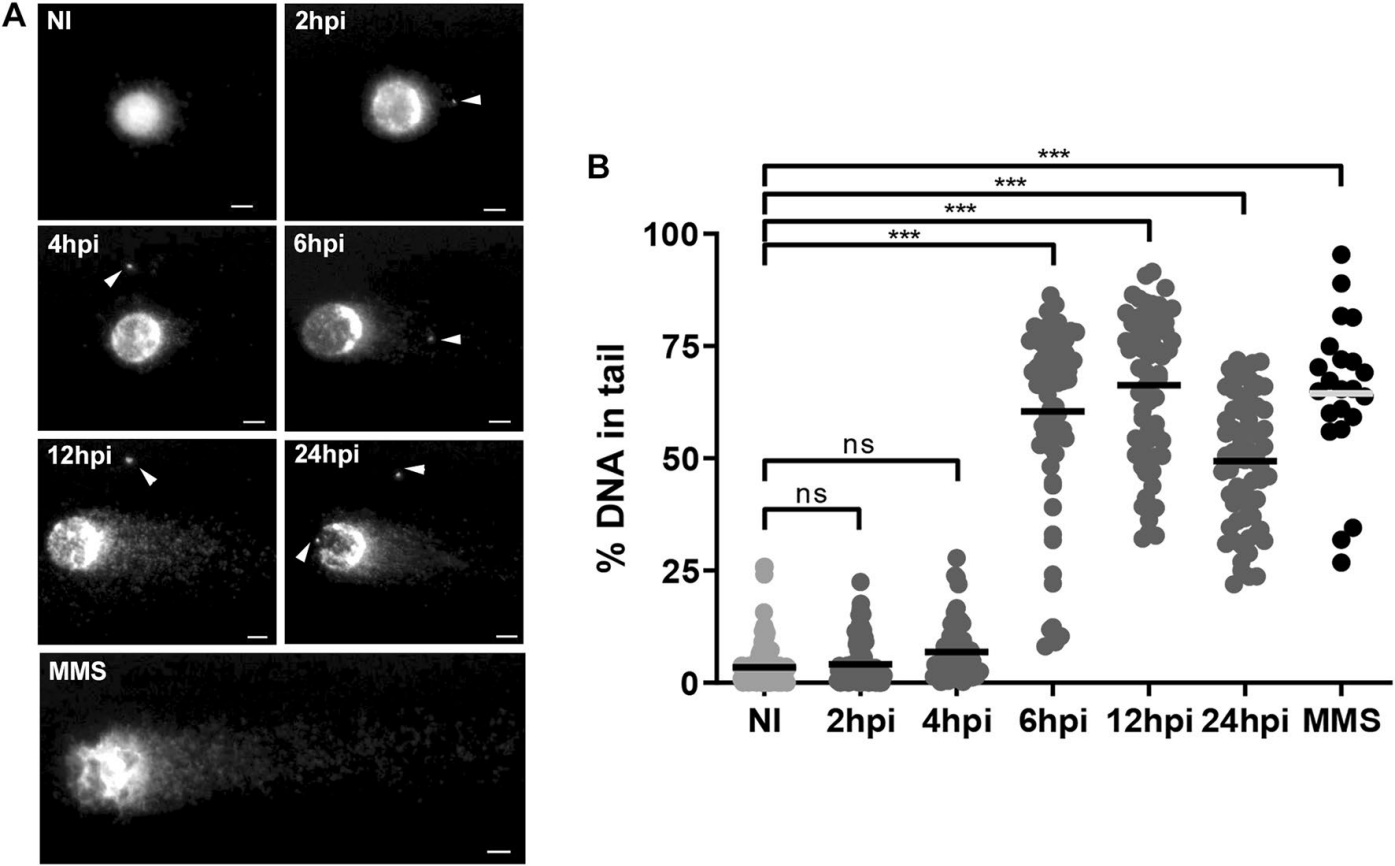
Comet assay shows DNA damage in infected LLC-MK2 cells by *T. cruzi*. **(A)** Images of comet assays of LLC-MK2 cells non-infected (NI) or infected with *T. cruzi* at different time points (2–24 hpi). Sybr Green I stained DNA in host cell nuclei and *T. cruzi* nuclei/kinetoplasts (arrowheads). *MMS* methyl methanesulfonate, used as a positive control for induction of DNA damage. Scale bar: 5 µm. **(B)** Quantification of fractured DNA fragments in the comet tails was done based by measuring the relative percentage (%) DNA between head and tail of each evaluated time (> 50 cells/time point, only cells with intracellular parasites were quantified). The bars represent the median value of the dataset of two independent experiments (n = 2). ***: (ρ < 0.001), ns: (ρ > 0.05) obtained by One-way ANOVA, and Dunn's test was used as a posthoc test in the GraphPad Prism v.8.0 software.

LLC-MK2 epithelial cell line has been shown to be susceptible to infection by different strains of *T. cruzi* providing advantages for studying host-parasite interactions and specific cellular and immune responses^42,43^. To assess the induction of DNA breaks in LLC-MK2 cells infected with *T. cruzi*, we performed the comet assay in the first 24hpi to measure strand breaks (% of DNA in the comet-tail) by exploiting the capacity of the broken DNA to migrate and form a tail when submitted to electrophoresis in the comet assay^44,45^. Our results show an increase in host DNA breaks at 6, 12, and 24 hpi, compared to non-infected (NI) cells (Fig. 1A). We observed fewer DNA breaks at 2 hpi and 4 hpi, but at 6hpi, the infected cells formed comet tails containing ~ 20 to 90% of the DNA content, suggesting an accumulation of DNA breaks (Fig. 1A, B). At 12hpi, we observed the highest value of median signal intensity (69%) among all the time points evaluated, indicating that the most significant DNA fragments in the comet tails occurred at this time. The amount of DNA fragments observed in the comet tail became reduced at 24 hpi, suggesting that after this time, some of these DNA breaks may have been resolved by the host cell (Fig. 1B).

### *T. cruzi* infection increases the phosphorylation of DNA damage sensor H2AX in LLC-MK2 cells in response to DSB

In eukaryotes, the variant histone H2AX is post-translationally modified at its residue S139 by ATM in response to DNA double-strand breaks (DSBs)^46,47^. The phospho-H2AX decorates sites of DNA lesions, stimulating the recruitment of other DDR proteins^48,49^.

**Figure 2:**
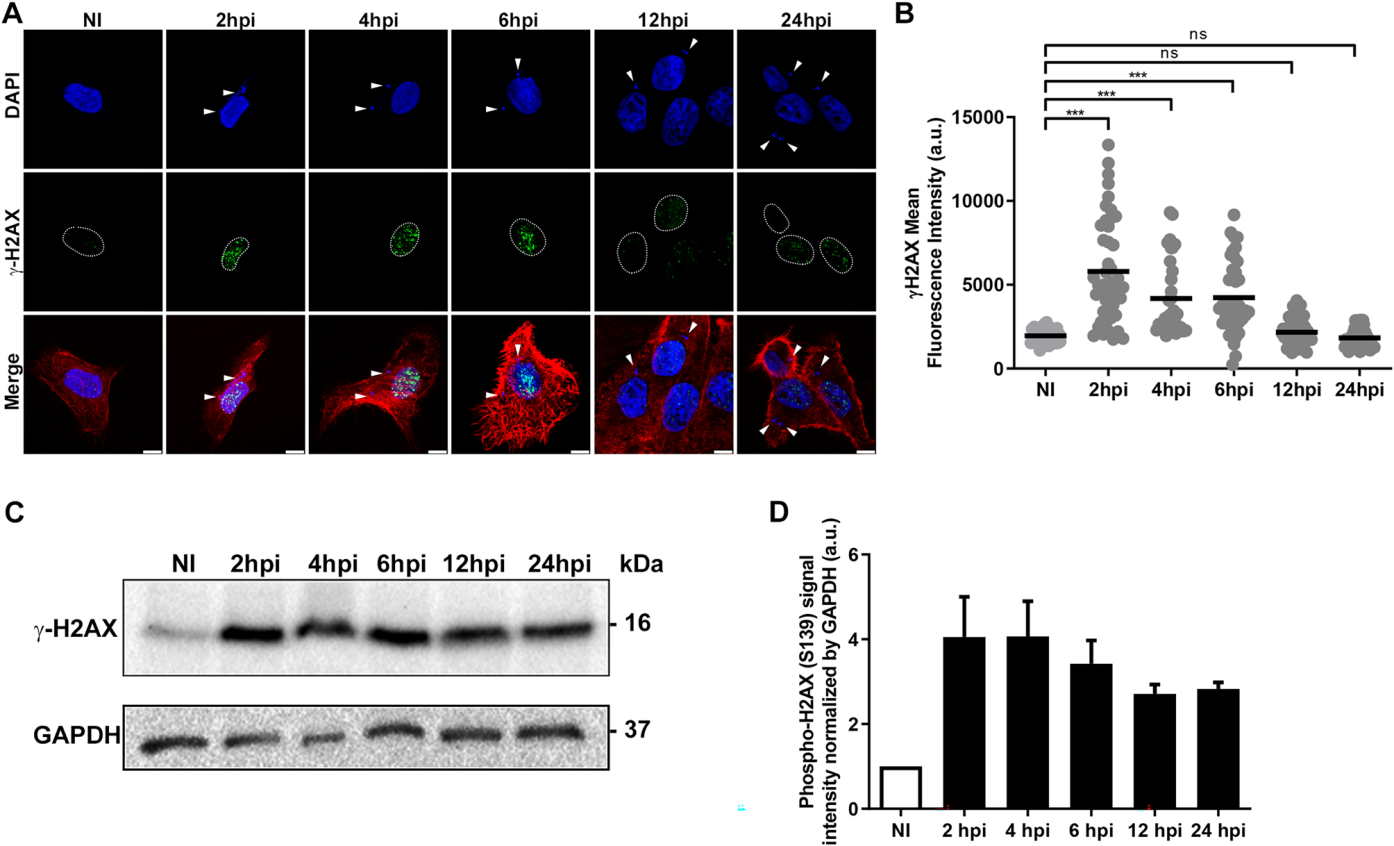
*Trypanosoma cruzi* infection induces rapid H2AX phosphorylation in LLC-MK2 cells in response to the DNA damage. **(A)** Confocal microscopy images of non-infected (NI) or *T. cruzi*-infected LLC-MK2 cells (2–24 hpi) are stained with anti-γ-H2AX antibody (green), demonstrating DNA damage in host cell nuclei. Actin is stained with rhodamin-phalloidin (red), host and parasite nuclei and kinetoplasts (arrowheads) are labelled with DAPI (blue). Host cell nuclei were delimited by a dotted line. Merged images are shown as indicated. Scale bar: 10 µm. **(B)** Quantification of the nuclear γ-H2AX fluorescence intensity of images acquired in epifluorescence microscopy during parasite progression of infection (n > 100 cells/time points) and analysed by ImageJ. Data presented are mean ± SEM, representative of two independent experiments (n = 2). ***: (ρ < 0.001) obtained by the One-way ANOVA test and the comparison performed by Dunnett as a posthoc test in the GraphPad Prism v.8.0 software. *a.u.* arbitrary units. Bars represent a mean value from the dataset. **(C)** Western Blot of LLC-MK2 cells lysates highlighting active levels of phospho-H2AX at different times of infection (2–24 hpi) compared to non-infected (NI) cells revealed with anti-γ-H2AX antibody. GAPDH was labelled with an anti-GAPDH antibody as endogenous control. Data are representative of two independent experiments (n = 2). *kDa* kilodaltons. **(D)** The bar graph represents the relative intensity bands in (**C**) normalized with the GAPDH protein.

To better understand the activation of the DDR pathway in LLC-MK2 cells infected with *T. cruzi*, we first examined the phosphorylation of H2AX at different times post-infection by immunofluorescence (Fig. 2). Confocal analysis revealed DNA damage foci in the nucleus-infected cells compared to NI cells (Fig. 2A). These results are similar to those observed in the DNA damage pattern of LLC-MK2 cells treated with 500 µM MMS, a potent chemical inducer of DNA damage^50^ (Supplementary Fig. S1). The quantification of total nuclear phospho-H2AX fluorescence intensity between 2 and 6 hpi showed a two-fold increase in DNA damage compared to NI cells (Fig. 2B). While phospho-H2AX foci were rapidly formed at 2hpi (Fig. 2A, B), comet assay detected lower levels of DNA breaks at this early time point as observed (Fig. 1). Furthermore, by immunoblotting, we detected higher levels of phospho-H2AX between 2 and 6hpi, which remained active up to 24hpi (Fig. 2C, D).

### 53BP1 and ATM are rapidly phosphorylated in response to the DNA damage in LLC-MK2 cells infected with *T. cruzi*

**Figure 3:**
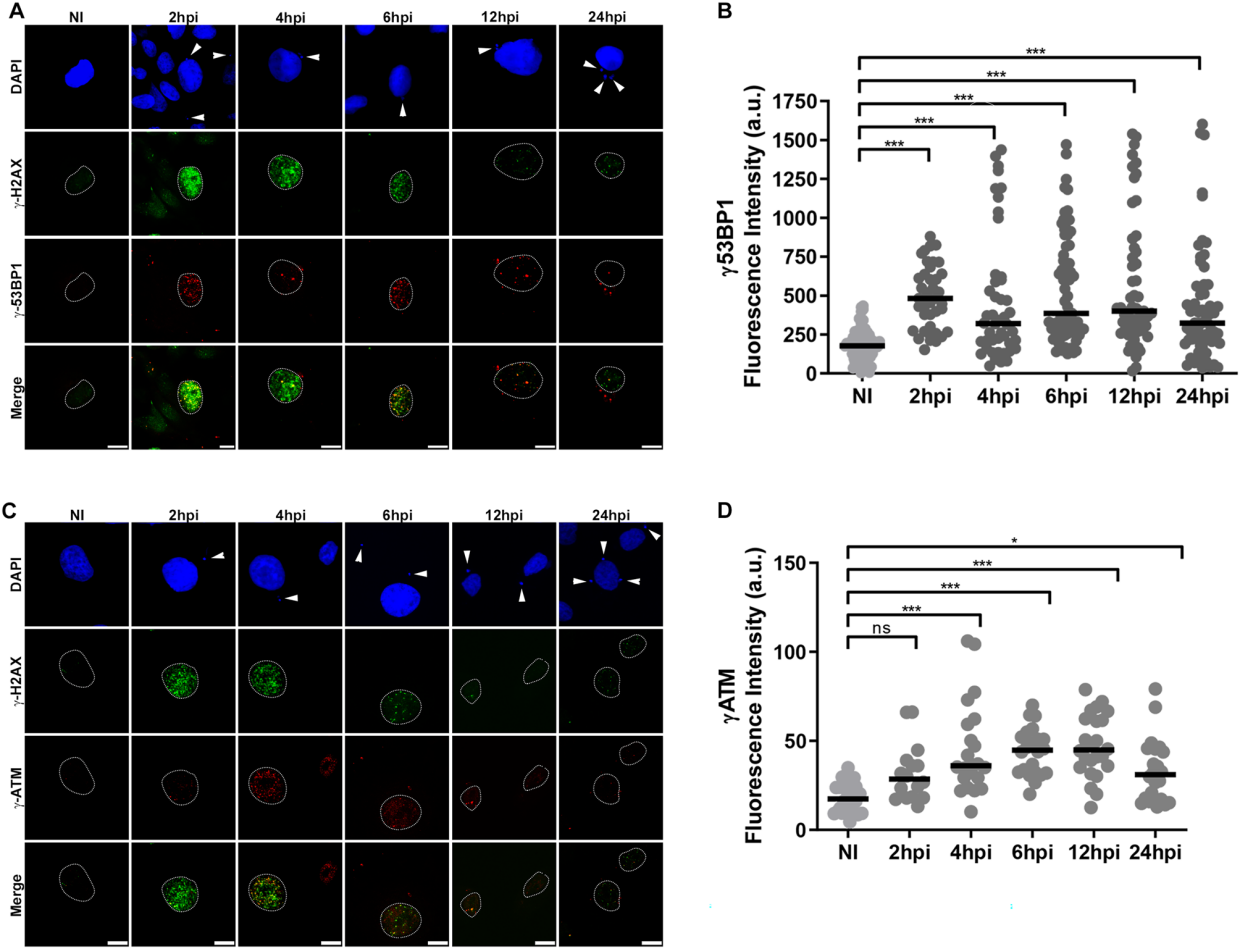
Phospho-53BP1 protein and phospho-ATM kinase are recruited rapidly to the nuclear foci in response to *T. cruzi* infection. **(A)** Confocal microscopy images are showing the localisation of phospho-53BP1 (red) and phospho-H2AX (green) in *T. cruzi*-infected LLC-MK2 cells at different time points (2–24 hpi). Host and parasite nuclei and kinetoplasts are stained with DAPI (blue). Arrowheads indicate parasites. Host cell nuclei were delimited by a dotted line. Merged images are shown as indicated. Scale bar: 10 µm. **(B)** Quantification of phospho-53BP1 nuclear fluorescence intensity acquired by epifluorescence microscopy during the progression of parasitic infection (n > 50 cells/time point). Data are representative of three independent experiments (n = 3). ***: (ρ < 0.001) obtained by the Kruskal–Wallis test and Dunn's multiple comparisons as a posthoc test in GraphPad Prism v.8.0 software. *a.u.* arbitrary units. The black bar shows the median value for each time point. **(C)** Confocal microscopy images of *T. cruzi*-infected LLC-MK2 cells (2–24 hpi) and NI cells showing the localisation of phospho-ATM (red) and phospho-H2AX (green) proteins in the cell nuclei. Host cell nuclei, parasite nuclei and kinetoplasts (arrowheads) were stained with DAPI (blue). Host cell nuclei were delimited by a dotted line. Merged images are shown as indicated. Scale bar: 10 µm. **(D)** Quantification of nuclear phospho-ATM fluorescence intensity in *T. cruzi*-infected cells obtained by epifluorescence microscopy compared to NI cells (n > 50 cells/time point). Black bars indicate the median value of the dataset. *a.u.* arbitrary units. ***: (ρ < 0.001), *: (ρ < 0.05), ns: (ρ > 0.05) obtained by the Kruskal–Wallis test and Dunn's multiple comparisons as a posthoc test in GraphPad Prism v.8.0 software. Data are representative of three independent experiments (n = 3).

To directly test DDR activation in *T. cruzi*-infected cells, we examined the phosphorylation of the mediator 53BP1 protein by confocal microscopy (Fig. 3). The formation of phospho-53BP1 foci in the host nucleus of infected cells was observed after 2hpi and remained active at all the analyzed time points (Fig. 3A, B). The double labelling immunofluorescence from 2 to 6 hpi revealed a co-localisation of phospho-53BP1 and phospho-H2AX in the host nucleus demonstrating the recruitment of the mediators to the DSB sites (Fig. 3A). The nuclear phospho-53BP1 fluorescence intensity analysis showed modulation of this phosphoprotein during the infection time, particularly, a 2.7-fold higher increase at 2 hpi compared to the non-infected (NI) cells (Fig. 3B). Furthermore, the immunoblotting analysis revealed an increase in the phosphorylation of 53BP1 during infection with the highest signal intensity at 6 hpi (1.6 times) compared to NI cells (Supplementary Fig. S2A), in accordance with the immunofluorescence results (Fig. 3A, B).

The 53BP1 protein is specifically phosphorylated at residue S1778 (phospho-53BP1) by ATM kinase^51^ and then interacts with phospho-H2AX to mediate the DDR pathway^52^. Then, to visualize phospho-ATM in infected cells, we performed a double immunofluorescence assay using an antibody that recognizes the serine residue at position 1981 (phospho-ATM) and phospho-H2AX. The confocal microscopy of infected cells showed the distribution of phospho-ATM foci in the nucleus coinciding with the higher phosphorylation of H2AX from 2 to 6 hpi (Fig. 3C). The nuclear phospho-ATM fluorescence intensity quantifications showed a significant increase from 4 hpi onwards, with intensities 2.5 times higher than NI cells at 6 and 12 hpi and decay at 24 hpi (Fig. 3D). Similarly, we observed an increase in the band intensity of phospho-ATM at 4, 6, and 24 hpi, as visualized by immunoblotting analysis (Supplementary Fig. S2B).

Active ATR kinase was analyzed by immunoblotting using an antibody to the phospho-threonine 1989 residue (phospho-ATR). We found comparable ATR phosphorylation levels among NI cells and infected cells, suggesting that this kinase is probably not necessarily associated with the *T. cruzi* infection (Supplementary Fig. S2C). As ATR is specific for DNA single-strand breaks (SSB) and DSB activates the ATM kinase^37,38,53^, our results suggest that *T. cruzi* infection may induce DSB in LLC-MK2 cells.

### The DSB repair pathway is activated and driven by DNA-PK kinase during *T. cruzi* infection

We showed that phospho-H2AX, phospho-53BP1, and phospho-ATM are activated in response to the DSB induction in the host cell. However, DNA damage is decreased at 24 hpi (Figs. 1, 2), suggesting that infected cells could resolve these DNA lesions.

In mammals, non-homologous end joining (NHEJ) and homologous recombination repair (HR) are known as major DSB repair pathways^54,55^. NHEJ is the principal repair pathway used by eukaryotes since it does not require a template strand, as with the HR repair pathway. While HR is only activated at S and G2/M phases, NHEJ can be activated in any cell cycle phase. Here, we investigated the key proteins of the repair pathways, DNA–protein kinase (DNA-PK) for NHEJ and Rad50 for HR.

**Figure 4:**
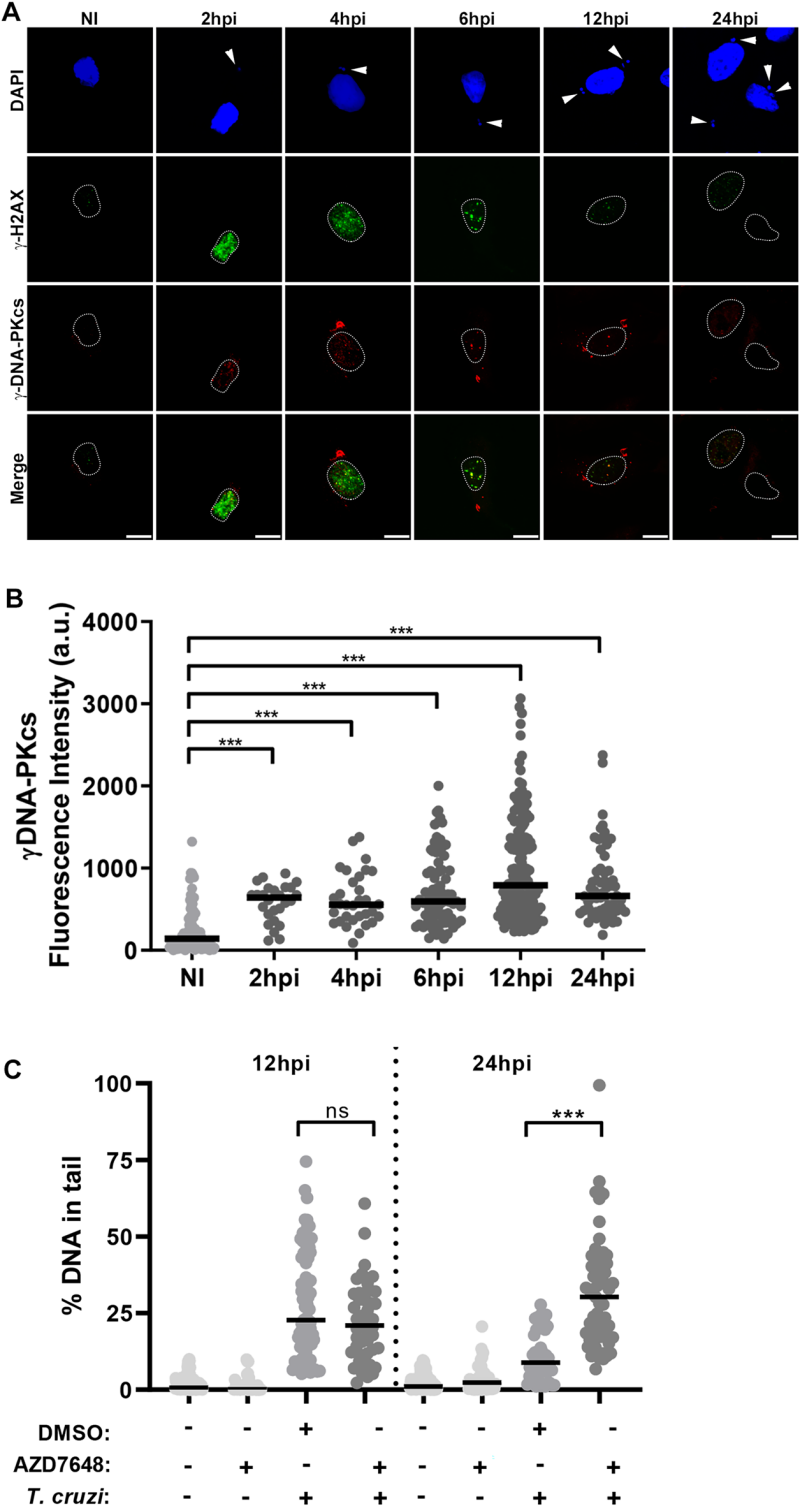
DSB repair is dependent by DNA-PK kinase during *T. cruzi* infection. **(A)** Confocal microscopy of *T. cruzi*-infected LLC-MK2 cells (2–24 hpi) and NI cells showing the localisation of phospho-DNA-PK (red) and phospho-H2AX (green) proteins. Host and parasite nuclei and kinetoplasts were stained with DAPI (blue). Host cell nuclei were delimited by a dotted line. Arrowheads indicate intracellular parasites. Merged images are shown as indicated. Scale bar: 10 µm. **(B)** Quantification of nuclear phospho-DNA-PK fluorescence intensity of *T. cruzi*-infected cells obtained by epifluorescence microscopy (n > 50 cells/time point). Data are representative of three independent experiments (n = 3). ***: (ρ < 0.001) obtained using the Kruskal–Wallis test and Dunn's multiple comparisons as a posthoc test in GraphPad Prism v.8.0 software. Black bars indicate the median value of the dataset. **(C)** Quantification of DNA damage by comet assay in LLC-MK2 cells that were either infected (+) or not (−) with *T. cruzi* (12 and 24 hpi), treated with the specific DNA-PK inhibitor (AZD7648) or with DMSO vehicle. The median value of each group is represented by the black bar. ns: (ρ > 0.05), ***: (ρ < 0.001). Statistical analysis was performed using the Kruskal–Wallis test followed by Dunn's multiple comparisons post hoc test in GraphPad Prism v.8.0 software.

DNA-PK is a member of the same PIKK family of kinases to which ATM and ATR belong^38^ and plays significant roles in repairing DSB via the NHEJ repair pathway^56^. The activation of DNA-PK may occur by phosphorylation at residue T2609 by ATM, which triggers rapid autophosphorylation of DNA-PK at residue S2056^57^. Double immunofluorescence labelling in NI and infected cells was done using an antibody that recognize the phosphorylation of DNA-PK at the S2056 residue (phospho-DNA-PK) and phospho-H2AX. Confocal microscopy showed the recruitment of the active form of DNA-PK (phospho-DNA-PK) to the DNA damage foci in the nucleus observed by the localization of the phospho-H2AX at different times of infection (Fig. 4A). Quantification of nuclear fluorescence intensity demonstrated that the first peak of phospho-DNA-PK activation occurs at 2hpi and significantly increased more than fivefold at 12hpi in infected cells compared to NI cells (Fig. 4B). By immunoblotting, we observed a high activity of phospho-DNA-PK at 12hpi compared to NI cells corroborating our immunofluorescence findings (Supplementary Fig. S2D).

We investigated whether Rad50, which participates in the HR repair pathway, could be activated during *T. cruzi* infection. Rad50 is phosphorylated by ATM at S635 residue (phospho-Rad50) and forms the Mre11-Rad50-Nbs1 (MRN) protein complex^54,58,59^. In contrast to DNA-PK (Fig. 4), no alterations were found in the phospho-Rad50 protein levels by immunoblotting during the parasite infection (Supplementary Fig. S2E).

To investigate whether DSB repair is depended on DNA-PK kinase activity, we treated cells with a potent and selective DNA-PK inhibitor, AZD7648^60^ and performed comet assay. After 12hpi, we observed that neither AZD7648 nor DMSO vehicle induced DNA damage in exposed LLC-MK2 cells (Fig. 4C). Remarkably, at 24hpi, cells exposed to AZD7648 exhibited greater DNA damage compared to control cells at the same time (Fig. 4C), thus confirming the role of DNA-PK kinase activation in DSB repair in host cells infected with *T. cruzi*.

Taken together, our data reveal that the DDR pathway is being orchestrated during *T. cruzi* infection through phosphorylation dynamics in a time-dependent perspective. Furthermore, the NHEJ repair pathway is activated and relies on DNA-PK kinase during infection to repair the induced DSB in order to maintain genome integrity.

## Discussion

Pathogens manipulate their host cell environment to ensure their survival, such as altering transcription^18,61,62^, translation^63^, metabolic and signalling pathways^64,65^, and inducing morphological modifications^13,14,66,67^. Pathogens can also directly or indirectly affect the host cell by inducing changes to the host cell DNA^30,31^ by the activation of the phosphoproteins H2AX, 53BP1, and ATM during the initial phase of the infection^21,23,26,68^. However, *T. cruzi* infections and their effects on host DNA remain poorly documented.

*Trypanosoma cruzi* evades the immune system by hiding within host cells and modulating the host immune response^6,8^. It is well known that *T. cruzi* infection correlates with an inactivation of the complement system^8^, activation of B cells to produce nonspecific antibodies^69^, a downregulation of T cells^7^, increased antioxidant capacity inside the PV of macrophages^70^ and infecting non-phagocytic cells in order to survive inside them^8^. Also, *T. cruzi* varies its antigenic surface proteins to avoid adaptive immune clearance^71^.

Our recent work demonstrated at the beginning of the infection, the parasite is closely associated with the host cell nucleus, causing a nuclear deformation, promoting RNA Pol II pausing in the host, altering host splicing, and sequestering a host spliceosome protein^18^. It is well known that nuclear deformation could affect chromatin organization, correlating with DNA breaks^72^. Here, we observed similar behaviour with parasites in close proximity to the host cell nucleus, suggesting a potential source of DNA injuries causing DNA breaks in the host. This event could be advantageous for parasite pathogenesis since DNA damage in infected cells could lead to regulation or interference in the host gene expression and transcription, as already seen in other studies^10,12,17,18,40,73,74^.

Transcriptome analyses of *T. cruzi*-infected cells revealed modulations in the fatty acid metabolic pathway, immune response, and cellular response to stress^75–77^. In addition, several proteins, including PARP-1 and XRRC6 were found upregulated in the host nucleus observed by proteomic analysis^41^. Here, we characterise the activation dynamics of the host key DDR pathway in LLC-MK2 cells, highlighting the initial response to DSB induced by *T. cruzi* infection. First, we used a comet assay to test for DNA breaks in *T. cruzi*-infected cells, finding from 6 to 12hpi, an accumulation and significant amount of fragmented DNA when compared to the NI cells (Fig. 1). Indeed, we found that at 24hpi, the infected cells exhibited a decrease in the amount of accumulated DNA breaks. Additionally, this assay is capable to show the capacity of the infected cells to resolve the DNA breaks^44^. Such results suggest that *T. cruzi* does not seem to prevent the host cell from repairing the DNA lesions that occurred during the first post-infection times. Thus, we decided to study the activation of the DNA damage response (DDR) pathway during *T. cruzi* infection. One of the initial signals in response to DNA damage is phosphorylation of the histone variant H2A, H2AX^46,48^, producing phospho-H2AX. We demonstrated that phospho-H2AX can act as a platform to recruit DDR proteins in response to DSB since we found phospho-H2AX signal ~ 3 fold higher at 2hpi in infected cells compared to NI cells. Furthermore, at 2hpi, we found 53BP1, ATM and DNA-PK rapidly phosphorylated, suggesting that the cell has experienced DNA injuries. The data obtained from the fluorescence intensity quantifications of *T. cruzi* infection and the activation of DDR proteins (Figs. 2B, 3B, D, 4B) can be seen in the graphical summary (Supplementary Fig. S3). Phospho-H2AX acts as a platform for hierarchical recruitment and retention of these phospho-proteins in a time-dependent manner during infection. These results are in agreement with the spatiotemporal recruitment of DDR proteins described by other authors^78–80^.

A marked activation of H2AX was observed at 2 hpi, as well as the recruitment of 53BP1 to the damaged site. The increase of phospho-53BP1 over time of infection coincides with the increase of phospho-ATM, as it is known that 53BP1 is activated by ATM, which becomes significantly active from 4 hpi onwards (Fig. 3). Maximum ATM phosphorylation, during *T. cruzi* infection in LLC-MK2 cells, from 6 to 12 hpi, indicating that ATM is activating other proteins downstream of the DDR pathway, corroborating the important role of the ATM protein in driving the DDR pathway in eukaryotes^38,57,81^.

In our study, we observed that DNA-PK is active at 2hpi, potentially responsible for the rapid upstream phosphorylation of H2AX, while the ATM protein remained activated, as demonstrated previously^82^. The peak of the DNA-PK activity at 12hpi (Supplementary Fig. S3) coincides with the highest amount of DNA fragments detected in the host observed by the comet assay. We also confirmed the critical repair role of this kinase through the NHEJ repair pathway, since its inhibition with AZD7648 led to an increased in the production of DNA breaks at 24 hpi (Fig. 4C).

In the NHEJ repair complex, DNA-PK is recruited after Ku70/80 heterodimers to stabilize broken DNA strands^56,83^. It has been shown during *T. cruzi* infection that Ku70 is increased in the nucleus at 6 hpi^41^. Together, our findings provide evidence that the assembly of the NHEJ complex, occurring between 6 and 12 hpi, then initiates the restoration of host DNA integrity. The dynamics of DNA-PK phosphorylation have also been documented in other studies involving virus-infected cells^84,85^. It is interesting to note that the significant accumulation of DNA breaks at 6hpi observed by the comet assay can be explained by the activity of the DNA-PK enzyme at 2 hpi which coincides with the repair of initial DNA breaks showing lower detection by the comet assay at 2 hpi and 4 hpi. Furthermore, it is known that the accumulation of intracellular reactive oxygen species (ROS) can induce DNA breaks in cellular models^86^. A previous study demonstrated that *T. cruzi* infection increases intracellular ROS production in the host during the initial hours of infection, with low levels observed at 3 hpi reaching maximum peaks between 6 and 12 hpi^61^. This finding could potentially explain our results with the low number of DNA breaks detected in the comet assay between 2 and 4 hpi and the significant increase observed at 6hpi (Fig. 1). Further studies are needed to explore and confirm these findings, shedding light on the intricate dynamics of DNA damage and repair during *T. cruzi* infection.

Previous studies have shown that the inhibition, blocking, and even degradation of RNA Pol II occurs when the DDR pathway is activated and mediated by ATM and DNA-PK^87^. We demonstrated that 12 hpi is a key moment of the host cell interaction with *T.* cruzi and at this time, RNA Pol II activity and the total RNA were drastically reduced in the host cell^18^. Our findings suggest that the response to putative DNA lesions correlating with *T. cruzi* infection is likely leading by ATM and the NHEJ repair pathway. The signalling cascade triggered by ATM and DNA-PK in response to the DSB may explain the quick shut-off of host transcription observed at 12 h during *T. cruzi* infection^18^ to ensure the efficiency and accuracy of repair of these lesions.

Here, we characterised the phosphorylation dynamics in a time-dependent manner of several key DDR proteins during *T. cruzi* infection. In this time window, DSB are repaired via the NHEJ pathway to protect the genome integrity. These findings will help to understand the strategies employed by *T. cruzi* to persist infection and may contribute to the search for effective therapeutic targets against this neglected disease.

## Materials and methods

### Antibodies and reagents

The antibodies used in this work include the following commercial antibodies: anti-phospho-histone H2AX (Ser139) (#80312-mouse), anti-phospho-53BP1 (Ser1778) (#2675-rabbit), anti-phospho-ATR (Thr1989) (#30632-rabbit), anti-phospho-Rad50 (Ser635) (#14223-rabbit) and anti-GAPDH (#3683-rabbit-HRP conjugated) from Cell Signaling Technology, USA. Other commercial antibodies include anti-phospho-(Ser/Thr) ATM/ATR Substrate (#AP0933-rabbit), anti-phospho-PRKDC (Ser2056) (#AP0621-rabbit) and anti-phospho-ATM (Ser1981) (#AP0008 -rabbit) from ABclonal, USA. Methyl methanesulfonate (MMS, Sigma, USA), DNA-PK inhibitor (AZD7648, AstraZeneca, UK), generously provided by Dr. Jennifer A. Black (Dept of Cellular and Molecular Biology and Pathogenic Bioagents, Ribeirão Preto Medical School, USP).

### Cell culture and parasite infection

The cell culture maintains and the infection process was according to Gachet-Castro et al.^18^ with little modifications. Briefly, LLC-MK2 cells were cultured and maintained in 25 cm^2^ treated culture flasks (Corning, USA) using a complete RPMI medium (Corning, USA) supplemented with 10% fetal bovine serum (FBS, Thermo Fisher Scientific, USA), 0.1 U/L penicillin, and 0.1 g/L streptomycin (Thermo Fisher Scientific, USA). *Trypanosoma cruzi* (Y strain) was propagated in LLC-MK2 cells and infective trypomastigotes were harvested and used to infect new cells^18^. For a positive control of DNA damage, non-infected LLC-MK2 cells where treated with 500 µM MMS^50^ for 3 h (Supplementary Fig. S1).

### Immunofluorescence and confocal microscopy

LLC-MK2 cells (4 × 10^4^ per well) were seeded in 24-well plates (Corning, USA) containing coverslips. After 24 h cells were infected with *T. cruzi* (MOI: 15) and collected at different hours post-infection (NI, 2, 4, 6, 12, and 24hpi). At every collection time point, wells were washed three times with PBS, and fixed for 15 min at room temperature (RT) with 4% paraformaldehyde (Electron Microscopy Science, USA) diluted in PBS. Subsequently, cells were permeabilized with 0.2% Triton X-100 (Sigma Aldrich, USA) diluted in PBS and blocked with blocking solution (2% bovine serum albumin (Sigma Aldrich, USA) diluted in PBS) for 15 min. Primary antibodies were diluted in blocking solution and incubated with cells for 30 min at RT, followed by three washes with PBS gently to avoid loss of cell adhesion. Next, cells were incubated with secondary antibodies conjugated to Alexa Fluor 488 or 594 (Life Technologies, USA) and Rhodamine-Phalloidin (Life Technologies, USA), both diluted in blocking solution at 1:600 for 30 min at RT. After three washes with PBS, coverslips were mounted on microscope slides with Fluoromount-G with DAPI (Invitrogen, USA).

### Immunofluorescence image analysis

The images were obtained through a Leica TCS-SP5 Confocal microscope with 63X oil immersion objective and detected with a Photomultiplier tube (PMT) and Hybrid detector (HyD) of the Multiuser Laboratory of Confocal Microscopy, Department of Cellular and Molecular Biology and Bioagents Pathogeny, Ribeirão Preto Medical School, University of São Paulo (FMRP/USP). The quantification analysis was done with images obtained from an epifluorescence microscope DMI 4000, with a 100× oil immersion objective (Department of Biochemistry and Immunology, FMRP/USP). Images were acquired in random fields up to a minimum of 50 up to a maximum of 200 infected cells per replicate. Fluorescence was quantified by ImageJ 1.49v (https://imagej.nih.gov/ij/), and data were statistically analyzed in GraphPad Prism v.8.0.

### Western blotting

LLC-MK2 cells (2 × 10^5^ per well) were seeded in 6-well plates (Corning, USA) and after 24 h infected with *T. cruzi* (MOI: 15) at 2, 4, 6, 12, and 24 h. Non-infected cells (NI) are used as control. At the time of collection, each well was washed three times with PBS, then, RIPA Buffer (50 mM Tris–HCl pH 8.0, 150 mM NaCl, 1% NP40, sodium deoxycholate 0.5% and 0.1% SDS) containing protease and phosphatase inhibitors (1 mM DTT, 1 mM benzamidine, 0.5 mM PMSF, 10 mM Na_3_VO_4_, 25 mM NaF), 2 mM MgCl_2_ and 1 mM DNase were added to the cells. Immediately, cells were scraped off, collected in a tube containing 1.4 mm diameter zirconium beads, and taken to the Precellys^®^ 24 homogenizer (Bertin Instruments, France) following the manufacturing protocol.

After lysis, samples were quantified in a UV/VIS BioSpectrometer (Eppendorf, Germany) and diluted in sample buffer (100 mM Tris, 4% SDS, 20% glycerol, 10% β-mercaptoethanol, 0.002% bromophenol blue and 4 M urea) and heated 5 min for 95 °C as described Baqui et al.^88^. Electrophoresis was performed in a homogeneous 12% polyacrylamide gel or a 2.5%-12.5% gradient gel^89,90^. We used as a protein marker: precision plus protein (Bio-Rad, USA) and rabbit skeletal muscle myofibrils^89,90^. Next, samples were transferred to a nitrocellulose membrane in the semi-dry or submerged system^18^. The membranes were stained with Ponceau, cut for specific antibody incubations. Then, after three washes with TBS-T (20 mM Tris, pH 8.0, 137 mM NaCl, and 0.05% Tween-20), membranes were blocked (3% BSA in TBS-T) for 2 h at room temperature (RT) and incubated with the primary antibodies overnight at 4 °C in gentle agitation. After that, three washes with TBS-T were made and the membrane was incubated with goat anti-mouse IgG or goat anti-rabbit IgG peroxidase-conjugated secondary antibodies for 50 min at RT. The signal was detected with enhanced chemiluminescence ECL Prime Western Blotting Detection Reagent (GE Healthcare, USA) according to the manufacturer’s instructions. The images were obtained through ChemiDoc XRS (Bio-Rad, USA) using ImageLab software, and the signal was quantified using ImageJ software, standardized according to the GAPDH protein, and analyzed by GraphPad Prism v.8.0.

### Comet assay

The comet assay or single cell gel electrophoresis assay was adapted from Collins^44^ and performed following the Minimum Information for Reporting on the Comet Assay (MIRCA) recommendations^91^. LLC-MK2 cells infected with *T cruzi* at different time points, non-infected cells and MMS treated cells were harvested and resuspended in 0.5% low melting point agarose at 37 °C and spread evenly over microscope slides previously coated with 1.5% agarose and incubated for 10 min at 4 °C to solidify. The slides were then incubated in lysis solution (2.5 M NaCl, 100 mM EDTA, 10 mM Tris, and 1% Triton X-100 at pH 10) for 24 h at 4 °C. After lysis, slides were incubated in alkaline electrophoresis buffer (1 mM EDTA and 300 mM NaOH, pH 13.0) for 20 min at 4 °C and submitted to electrophoresis (300 mA and 25 V) for 20 min. Then, slides were placed in a neutralization buffer (0.4 M Tris pH 7.5) at 4 °C for 15 min and dried at RT for 20 min. Next, slides were fixed in absolute ethanol for 3 min and DNA was incubated with SybrGreen I (Sigma-Aldrich, USA) and analyzed immediately by Comet IV software (Instem, USA) at the Department of Genetics, FMRP/USP.

To assess the role of DNA-PK in DSB repair, we treated LLC-MK2 cells with 1 µM DNA-PK inhibitor (AZD7648) or DMSO vehicle for one hour before infecting with *T. cruzi* (12 and 24hpi) or without infection^60^.

### Statistical analysis

Results are presented as mean ± standard error of the mean (SEM) or median and interquartile range. Data were analyzed using Prism v.8.0 software (GraphPad Software Inc., La Jolla, CA, USA). All statistical determinations of normality were analyzed using the D'Agostino & Pearson test. The one-way analysis of variance (ANOVA) test was applied together with a post-test of Dunnett's multiple comparisons and, when the distribution was not assumed to be normal, the Kruskal–Wallis test was used in conjunction with a post-test of Dunn's multiple comparisons. In all analyses, infected groups were compared to the non-infected groups. Differences that provided ρ < 0.05 were considered statistically significant. All experiments were performed in biological duplicate (n = 2) or triplicate (n = 3).

### Supplementary Information

Supplementary Legends.

Supplementary Figures.

## Data Availability

All data generated or analyzed during this study are included in this published article or supplementary file. All other data that support the findings of this study are available from the corresponding author on reasonable request.
