# Supplementary material for: Trypanosoma cruzi infection induces DNA double-strand breaks and activates DNA damage response pathway in host epithelial cells
Source: Sci Rep. 2024 Mar 4;14:5225. doi: 10.1038/s41598-024-53589-w (PMC10909859; doi:10.1038/s41598-024-53589-w)
Supplement: Supplementary file 1 — Supplementary Legends. [file 41598_2024_53589_MOESM1_ESM.pdf]

Supplementary Information legends:

Supplementary Figure S1: The effect of methyl methanesulfonate (MMS) on LLC-MK2 cells. LLC-MK2 cells were incubated with 500 µM MMS for three hours or without (control). DDR pathway proteins were verified with anti-phospho-H2AX, -phospho-53BP1, -phospho-ATM and -phospho-DNA-PK antibodies (all in red). Cell nuclei were stained with DAPI (blue). The merged images are displayed as indicated. Bar: 10 µm.

Supplementary Figure S2: Activation of DDR pathway proteins in LLC-MK2 cells Infected with *T. cruzi*. (A-E) Western blotting quantification of phospho-53BP1, phospho-ATM, phospho-ATR, phospho-DNA-PK, and phospho-Rad50 proteins in NI and *T. cruzi*-infected cells respectively. Bar graphs represent the relative intensity of bands normalized by GAPDH protein. Data are based on three independent experiments (n=3).

Supplementary Figure S3: Summary of the activation dynamics of DDR pathway proteins during *T. cruzi* infection. The graphical depiction of the fluorescence quantification data (Figures 2B, 3B, 3D, 4B) shows the activation dynamics of DDR pathway proteins during *T. cruzi* infection. At 2hpi, we observed the activation of H2AX, 53BP1, and DNA-PK proteins, and, at 12hpi, we observed a highest activation of ATM and DNA-PK kinases (highlighted areas). Analyzes were performed using GraphPad Prism v.8.0 software.

Supplementary Figure S4: Full-Length Western Blot membranes. Representative detection of **(A)** phospho-H2AX and GAPDH (Fig. 2C); **(B)** phospho-53BP1, **(C)** phospho-ATM, **(D)** phospho-ATR, **(E)** phospho-DNA-PK, **(F)** phospho-Rad50, and GAPDH proteins (Fig. S2) in non-infected (NI) and infected LLC-MK2 cells with *T. cruzi*. Membranes were stained with Ponceau (full membranes), then cut for specific antibody incubations (cut membranes). It is important to note that all parts of the membranes are complementary. See material and methods for western blotting details.
