# Supplementary figures and images for: Trypanosoma cruzi infection induces DNA double-strand breaks and activates DNA damage response pathway in host epithelial cells
Source: Sci Rep. 2024 Mar 4;14:5225. doi: 10.1038/s41598-024-53589-w (PMC10909859; doi:10.1038/s41598-024-53589-w)

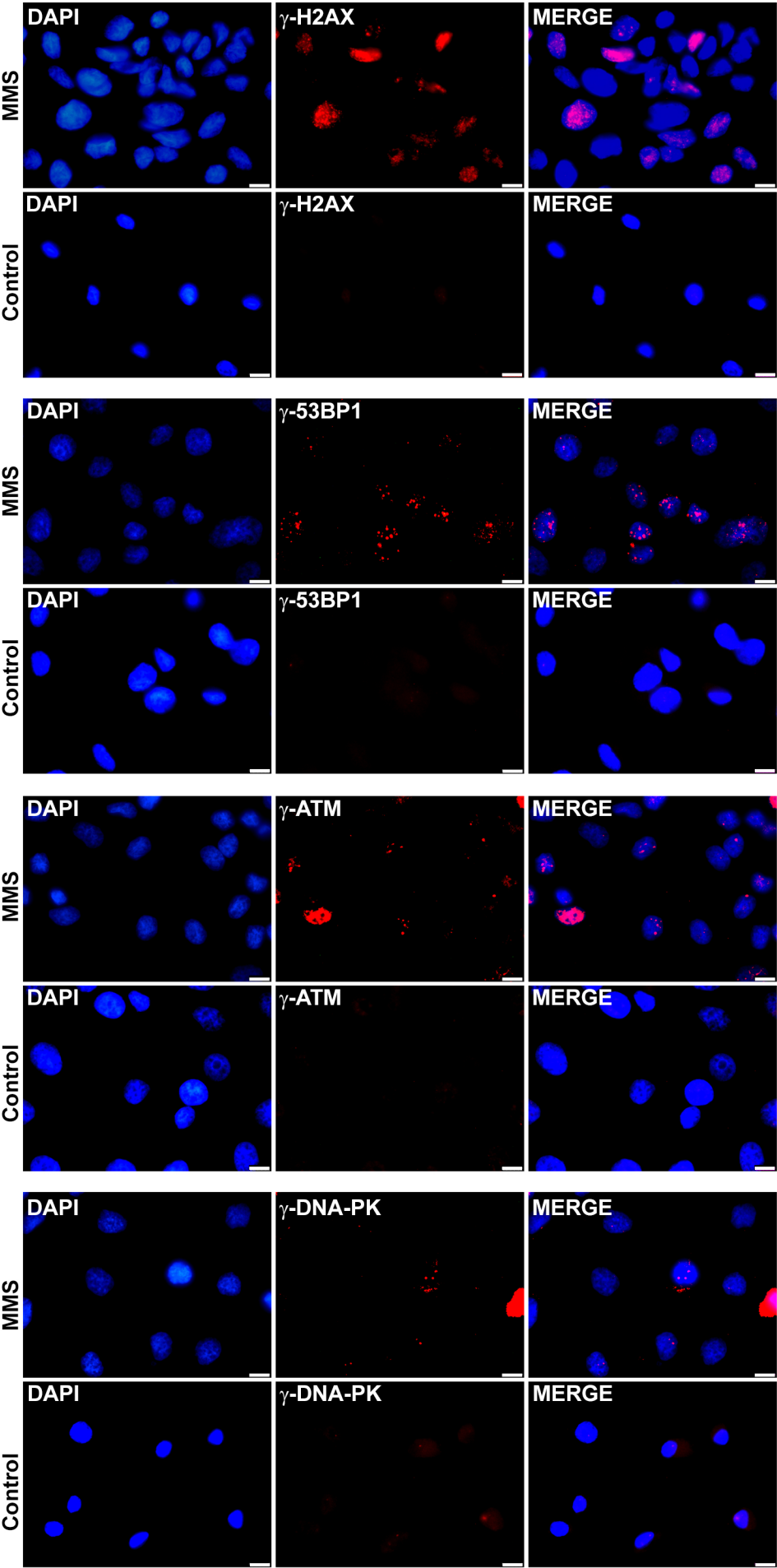

**A**

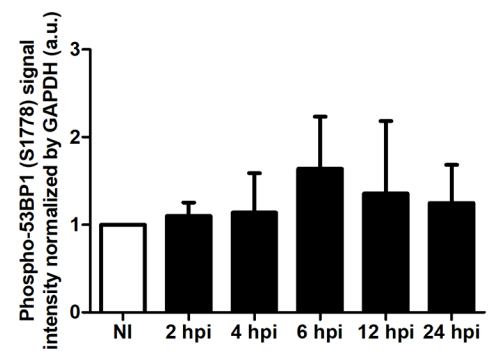

**B**

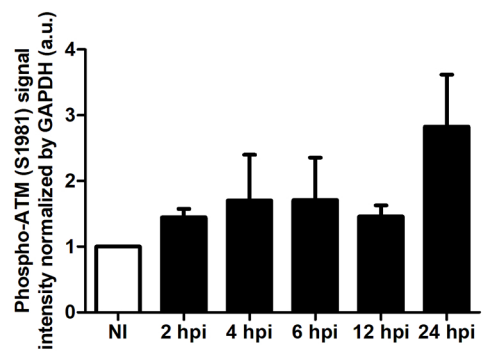

**C**

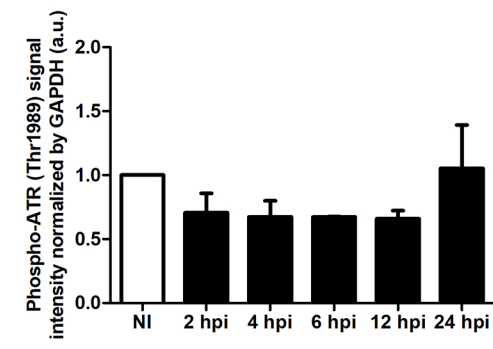

**D**

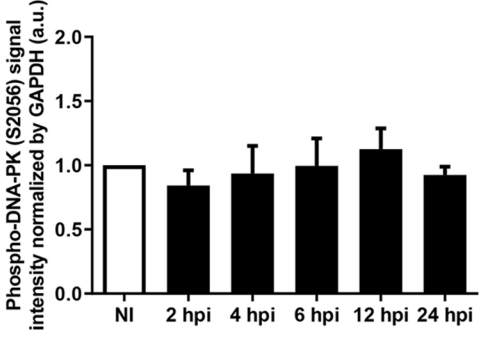

**E**

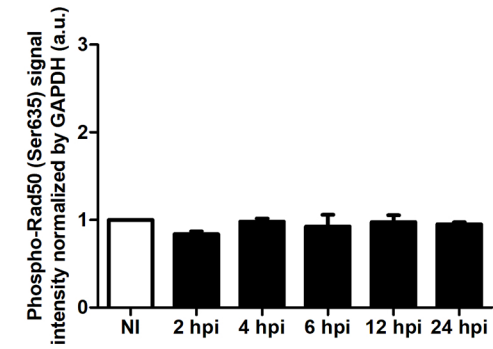

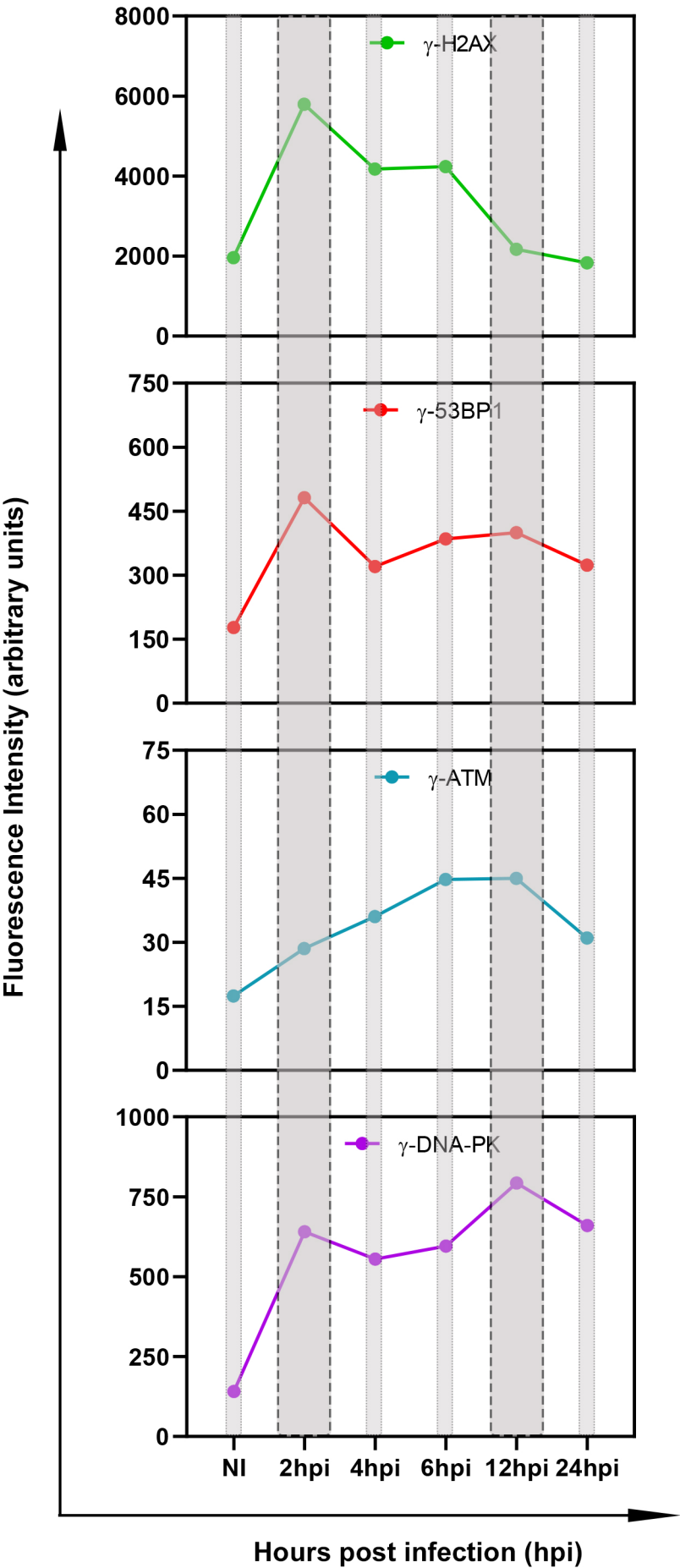

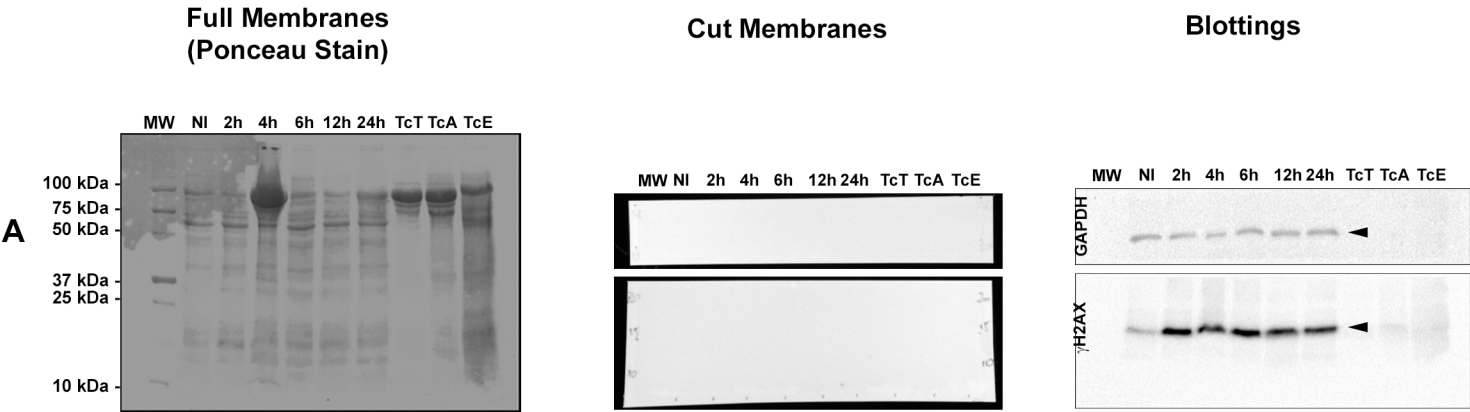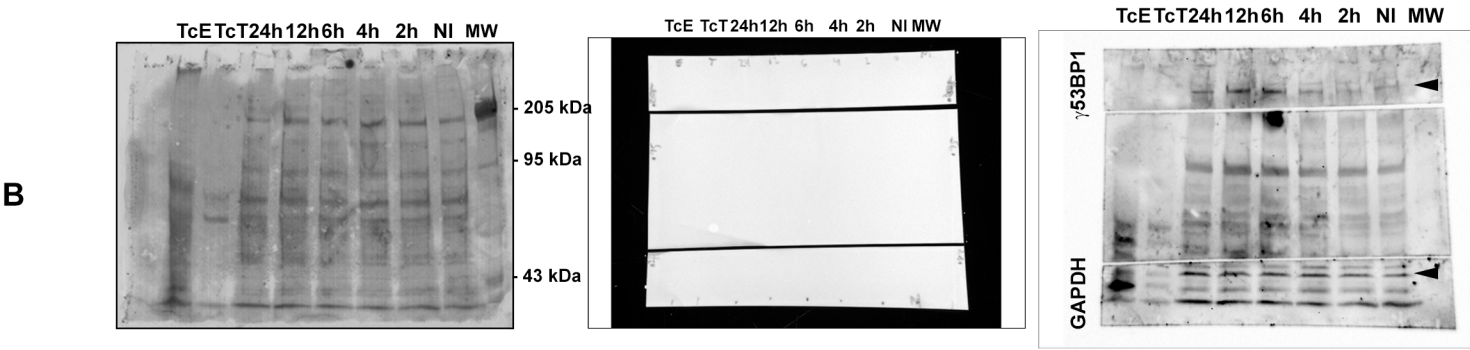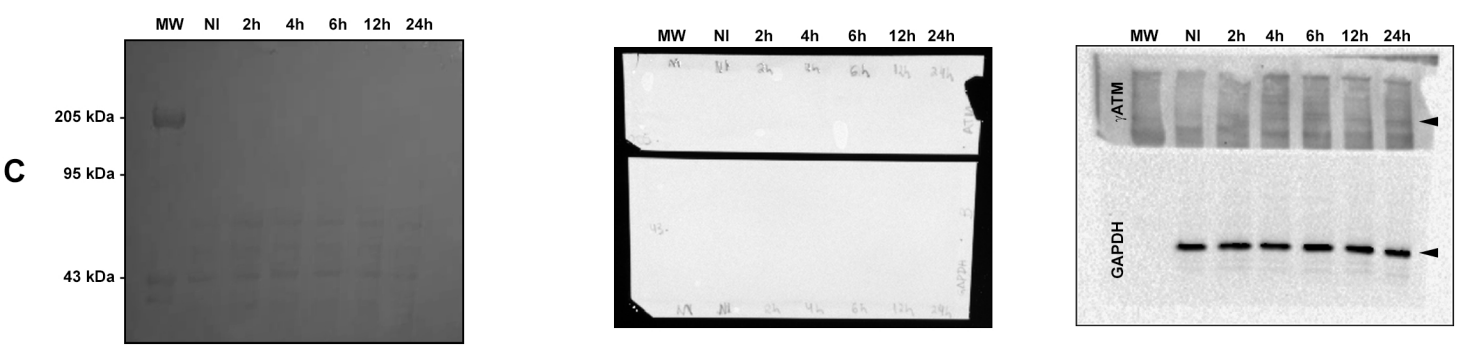

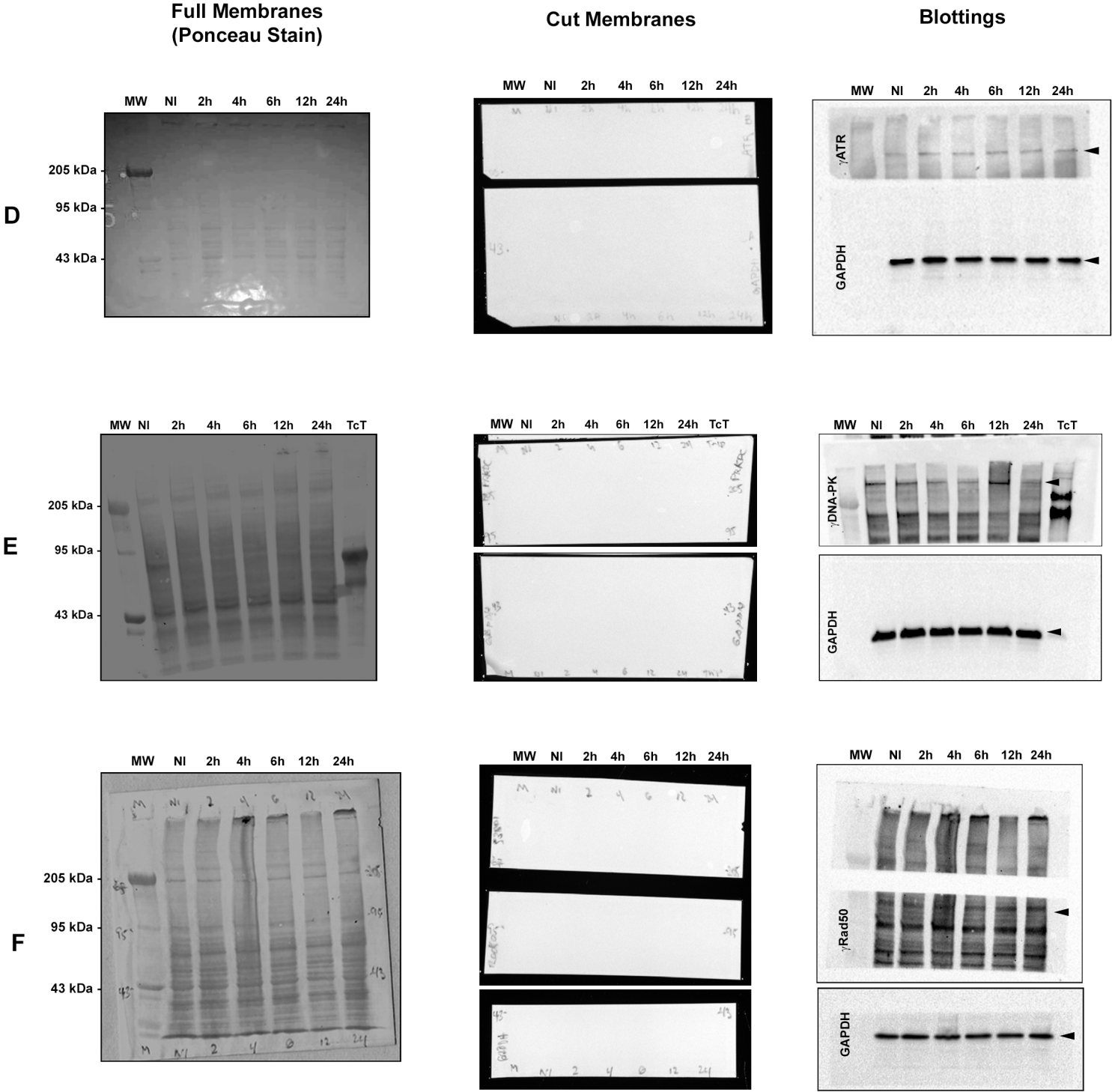

Supplement: Supplementary file 2 — Supplementary Figures. [file 41598_2024_53589_MOESM2_ESM.pdf]
